# Quantifying Erythema and Skin Tone Variation in Cutaneous Rashes: A Colorimetric Approach

**DOI:** 10.7759/cureus.107251

**Published:** 2026-04-17

**Authors:** Kawaiola Cael Aoki, Summer Wong, Simona Bartos, Harvey N Mayrovitz

**Affiliations:** 1 Medicine, Nova Southeastern University Dr. Kiran C. Patel College of Osteopathic Medicine, Fort Lauderdale, USA; 2 Dermatology, Memorial Healthcare, Pembroke, USA; 3 Dermatology, Imperial Dermatology, Hollywood, USA; 4 Medical Education, Nova Southeastern University Dr. Kiran C. Patel College of Allopathic Medicine, Davie, USA

**Keywords:** colorimetry, dermatology, diagnostic accuracy, erythema, fitzpatrick phototype, individual typology angle (ita), inflammation, melanin, pigmentation, skin of color

## Abstract

Background: Accurate erythema assessment is essential in dermatologic evaluation, yet detecting erythema in diverse skin tones remains challenging. Traditional visual assessment methods, including the Fitzpatrick Skin Type Classification Scale, have limitations in darker skin tones, where erythema may be underestimated or present differently. Objective colorimetric tools, such as the SkinPhotoTyper and SkinColorCatch from Delfin Technologies Ltd, Kuopio, Finland, provide quantitative measurements of erythema and pigmentation; however, their performance across diverse skin tones remains underexplored.

Methods: A cross-sectional study was conducted among 44 patients with rashes attending an outpatient dermatology clinic. Erythema index (EI), melanin index (MI), Individual Typology Angle (ITA), and CIELAB (Commission Internationale de l’Éclairage L*a*b*) values were measured at unaffected (baseline) and affected (rash) sites using calibrated colorimetric devices. Paired t-tests compared baseline and rash measurements, Pearson correlations evaluated relationships among colorimetric parameters, and one-way ANOVA examined differences across Fitzpatrick phototypes, ITA categories, rash locations, and rash types.

Results: As anticipated, erythema was elevated in rash sites compared to baseline, and higher melanin correlated with lower ITA and lightness. Unexpectedly, baseline erythema did not correlate with rash erythema, and darker ITA categories demonstrated higher erythema levels, suggesting that localized inflammation may not be accurately reflected by baseline skin tone or visual assessment alone.

Conclusion: This study supports the use of colorimetric tools across skin tones and highlights their potential in clinical assessment. Future research should focus on larger, longitudinal studies to evaluate changes in erythema and pigmentation during treatment. Such work could improve diagnostic accuracy, personalize treatment, and promote equity in dermatologic care, particularly by improving disease severity assessment, treatment decisions, and monitoring in conditions where erythema is less visually apparent.

## Introduction

Dermatology heavily relies on the visual assessment of skin tone and erythema as foundational elements in diagnosing and managing skin conditions. The Fitzpatrick Skin Type Classification Scale has traditionally facilitated this process, categorizing skin types based on their response to sun exposure [1]. However, recent research highlights significant limitations of this approach, particularly in capturing the nuances of skin tone diversity [2]. Developed with a focus on individuals of European heritage, the Fitzpatrick Scale often fails to accurately classify individuals with darker skin tones, leading to misclassification and potentially compromised care [3]. Furthermore, challenges in accurately detecting erythema in skin of color are well-documented, as the manifestations of erythema can differ significantly from those observed in lighter skin tones [2,4]. This is particularly evident in dermatologic conditions such as atopic dermatitis, rosacea, and psoriasis, in which misdiagnosis or delayed diagnosis can exacerbate patient outcomes [5-7].

Erythema is a critical feature in dermatologic diseases as an indicator of inflammation, but it is notoriously challenging to diagnose in individuals with skin of color [7,8]. For example, atopic dermatitis, which typically presents as recurrent pruritic papules and plaques, exhibits considerable variability in morphology, distribution, texture, and pigmentation across racial and ethnic groups. In skin of color, erythema may appear violaceous or brown, complicating its detection and potentially delaying diagnosis and treatment [5]. Similarly, in rosacea, challenges in recognizing erythema and telangiectasia in darker skin tones have contributed to underreporting, misdiagnosis, and inadequate treatment, leading to greater morbidity [6]. For psoriasis, detecting erythema is particularly problematic, as lesions that appear pink or red in lighter skin often present as violaceous or hyperpigmented in darker skin. This variability increases the potential for misinterpreting active psoriatic lesions as post-inflammatory hyperpigmentation, especially when minimal thickening and scaling are present [7,9].

Recognizing these challenges, there is a growing need for a more inclusive and objective approach to skin assessment in dermatology [10]. Technological advancements in colorimetric measurement tools, such as the hand-held compact SkinPhotoTyper (SPT) and SkinColorCatch (SCC) from Delfin Technologies Ltd, Kuopio, Finland, provide promising solutions by employing three-dimensional color spaces to enhance the precision and reproducibility of skin tone and erythema evaluation. Furthermore, integrating the individual typology angle (ITA) as an alternative classification system offers a more nuanced and inclusive framework for assessing skin tone, moving beyond the constraints of the Fitzpatrick Scale and further advancing precision in dermatological and cosmetic science [10,11].

This study’s primary objectives were exploratory: (1) to characterize the range of erythema across Fitzpatrick phototypes, (2) to examine the relationship between erythema and melanin content, and (3) to evaluate the performance of colorimetric devices (SPT and SCC) in detecting erythema across skin tones. The study employs a cross-sectional design to address these objectives, enrolling participants with rashes, including atopic dermatitis, psoriasis, acne, pityriasis rosea, and granuloma annulare, at an outpatient clinic in a diverse metropolitan area.

This study aims to contribute to a more inclusive and precise understanding of skin health by addressing the challenges of erythema detection and embracing technological innovations. Ultimately, this study seeks to advance dermatologic care, particularly in populations with skin of color, by fostering equity and precision across diverse patient populations.

## Materials and methods

Study design and participants

This cross-sectional study was conducted at Imperial Dermatology, an outpatient dermatology clinic in Hollywood, Florida. The study was approved by the Nova Southeastern University Institutional Review Board (IRB #2024-58-NSU). Patients 18 years and older seeking treatment for rashes were invited to participate following their dermatology visit. No restrictions were placed on rash severity or prior treatment. A total of 44 participants met the inclusion criteria, provided informed consent, and were enrolled in the study.

Description of devices

The SPT utilizes high-resolution imaging under standardized lighting conditions to classify skin type based on the Fitzpatrick Scale, analyzing melanin concentration through red, green, and blue (RGB) color values and lightness, green-red, and blue-yellow (L*a*b*) color space. By comparing these values against a validated reference database, the SPT facilitates an objective assessment of sunburn susceptibility, pigmentation disorders, and therapeutic responses to phototherapy. Similarly, the SCC employs spectrophotometry to emit controlled light onto the skin and measure its reflectance, allowing quantification of melanin and hemoglobin levels. These devices mitigate the subjectivity and inconsistencies associated with traditional visual assessments by converting reflectance data into RGB and L*a*b* color coordinates, thereby enhancing the accuracy of skin tone classification for dermatological research, cosmetic science, and personalized skincare formulations.

Data collection and measurement procedures

Measurements were performed immediately following the clinical encounter in a consistent clinical setting under stable ambient lighting. Participants were seated and allowed brief acclimatization prior to data collection, and lotions were removed before measurement. Devices were used and calibrated according to manufacturer recommendations, with consistent probe contact applied without excessive pressure.

For each participant, two measurement sites were selected and measured. The inner bicep of one arm (non-sun-exposed) was measured first. The arm was chosen based on patient preference. This location was used to assess the patient’s intrinsic phototype using the SPT and the melanin index (MI), erythema index (EI), and ITA using the SCC. Then, a rash area identified as the most clinically erythematous region by the operator was measured using the SCC. Only one lesion per patient was assessed. The SPT was not used at the rash site as it is designed solely for phototype classification. 

All measurements were performed by a single trained operator to minimize inter-operator variability. Single measurements were obtained at each site and recorded at the time of collection for subsequent analysis.

Statistical analysis

All statistical analyses were conducted using IBM SPSS Statistics for Windows, version 29 (IBM Corp., Armonk, NY, USA). To evaluate the differences between bicep and rash areas, paired samples t-tests were performed on EI, MI, ITA, and color dimensions (L*, a*, b* values). Correlation analyses examined the relationships between bicep and rash characteristics for each of the above parameters. Normality of continuous variables was assessed using Shapiro-Wilk testing and visual inspection of Q-Q plots. Although some variables demonstrated deviations from normality, parametric tests were retained because the sample size exceeded 30 observations and parametric methods are considered robust under these conditions. In addition, the primary analyses were based on paired within-subject comparisons (paired samples t-test), which further reduces the influence of distributional assumptions. The primary outcome was the within-subject difference in erythema index (EI) between baseline (bicep) and rash sites.

The ITA categories were classified according to the reference ITA skin classification system as described by Osto et al. (2022), where ITA angles were categorized as follows: Very Light (ITA° > 55), Light (41 < ITA° ≤ 55), Intermediate (28 < ITA° ≤ 41), Tan (10 < ITA° ≤ 28), Brown (-30 < ITA° ≤ 10), and Dark (ITA° < -30) [11].

A one-way analysis of variance (ANOVA) was conducted with independent variables, including phototype, ITA categories, anatomical rash location, and rash type, to assess variations in skin characteristics across different categorical groups. Significant ANOVA results were further analyzed using post hoc tests (Tukey HSD) to identify specific pairwise differences between groups. Statistical significance was set at p < 0.05 for all analyses. Unless specified otherwise, data are presented as mean ± SD.

Participant enrollment was based on feasibility and the number of eligible patients presenting during the study period. Because this study was exploratory and no prior literature provided effect size estimates for the primary paired colorimetric outcomes between baseline and rash sites, a formal a priori power calculation was not performed. To contextualize the achieved sample size, a post hoc sensitivity analysis was performed. With 44 participants, the study had approximately 80% power to detect moderate paired effect sizes (Cohen’s d ~ 0.44) and moderate correlations (r ~ 0.42) at α = 0.05 (two-sided). This study was designed as an exploratory observational analysis to generate preliminary effect size estimates to inform adequately powered future studies.

## Results

Demographic and descriptive statistics

**Figure 1:**
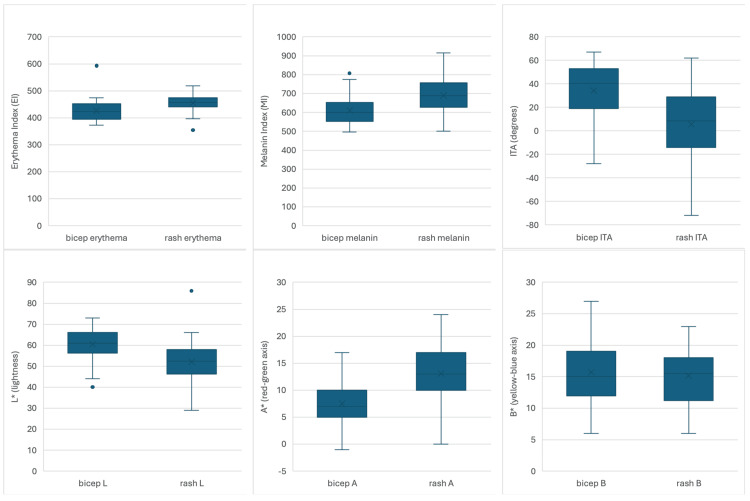
Distribution of baseline and rash colorimetric parameters Boxplots illustrating the distribution of colorimetric parameters measured at baseline (bicep) and rash sites (n = 44). Top row (left to right): erythema index (EI), melanin index (MI), and Individual Typology Angle (ITA). Bottom row: CIELAB color dimensions L* (lightness), A* (red–green axis), and B* (yellow–blue axis). Boxes represent the interquartile range with the median indicated by the horizontal line, and points represent outliers. Although baseline and rash measurements demonstrate overlapping ranges due to inter-individual biological variability, paired within-subject analyses revealed significant paired differences in EI, MI, ITA, L*, and A* (all p < 0.001).

The study sample consisted of 44 participants (19 to 91 years, mean 44.8 ± 21.4). The distribution of baseline and rash colorimetric parameters is illustrated in Figure 1, and the quantitative values are in Table 1.

**Table 1:** Average baseline and rash characteristics ITA = Individual Typology Angle; L, A, B = CIELAB color dimensions (L* = lightness, A* = red–green axis, B* = yellow–blue axis).

Variables	Baseline		Rash	
	Mean ± SD	Range	Mean ± SD	Range
Age	44.8 ± 21.4	19 - 91	-	-
Erythema	425.9 ± 38.5	372 - 593	455.0 ± 28.0	355 - 519
Melanin	610.7 ± 75.1	497 - 807	690.1 ± 95.9	501 - 916
ITA	34.4 ± 22.7	-28 - 67	5.7 ± 26.2	-72 - 62
L*	60.6 ± 7.4	40 - 73	52.1 ± 9.7	29 - 86
A*	7.5 ± 3.9	-1 - 17	13.1 ± 4.6	0 - 24
B*	15.7 ± 5.0	6 - 27	15.2 ± 4.5	6 - 23

Rash areas showed higher erythema (455 ± 28) than baseline (426 ± 39). Melanin levels were also higher in rash areas (690 ± 96 vs. 611 ± 75). Rash ITA was lower (5.7 ± 26.2 vs 34.4 ± 22.7), consistent with darker pigmentation. Colorimetry confirmed lower lightness (52.1 vs 60.5) and higher redness (13.1 vs 7.5) in rash areas, while yellowness (B*) did not differ significantly. Effect sizes ranged from moderate to large (Cohen’s d = 0.58-1.32), indicating that the observed differences reflect meaningful within-subject changes. Baseline skin characteristics are presented in Table 2. Phototype and ITA classify skin into six and five categories, respectively. Few patients in this study had darker skin tones, specifically Phototype 6 (n = 1) and Brown ITA (n = 7). The largest groups consisted of Phototypes 2 and 3 (n = 10 for both) and Light ITA (n = 14).

**Table 2:** Categorical baseline skin characteristics ITA = Individual Typology Angle. ITA categories were defined as: Very Light (ITA° > 55), Light (41 < ITA° ≤ 55), Intermediate (28 < ITA° ≤ 41), Tan (10 < ITA° ≤ 28), Brown (-30 < ITA° ≤ 10), and Dark (ITA° < -30).

Category		Frequency	Percent
Phototype	1	9	20.5
	2	10	22.7
	3	10	22.7
	4	7	15.9
	5	7	15.9
	6	1	2.3
	Total	44	100.0
ITA category	Very light	7	15.9
	Light	14	31.8
	Intermediate	8	18.2
	Tan	8	18.2
	Brown	7	15.9
	Total	44	100.0

Baseline ANOVA results across phototype groups and ITA categories

Significant differences were observed across phototype groups. Baseline EI levels varied significantly among phototypes (p = 0.004), indicating that EI levels are influenced by skin type. Consistent with expected pigmentation gradients, melanin levels and ITA values differed significantly across phototypes (p < 0.001 for both). Significant differences were further identified in skin coloration parameters, including lightness (L*), redness (A*), and yellowness (B*) (p < 0.001 for all).

Significant differences were also observed across ITA categories. EI (p = 0.003), melanin (p < 0.001), ITA values (p < 0.001), and lightness (L*) (p < 0.001) all varied significantly between categories. Differences were further identified in redness (A*) (p = 0.012) and yellowness (B*) (p < 0.001). ANOVA results are summarized in Table 3.

**Table 3:** Baseline skin characteristics across phototype and ITA category groups ITA = ITA = Individual Typology Angle; L, A, B = CIELAB color dimensions (L* = lightness, A* = red–green axis, B* = yellow–blue axis). One-way ANOVA (F-test) was used to evaluate differences in baseline colorimetric parameters across Fitzpatrick phototype groups and ITA skin tone categories.

Variable	Phototype F (df=5,38)	p-value	ITA category F (df=4,39)	p-value
Erythema index	4.218	0.004	4.723	0.003
Melanin index	56.159	<0.001	63.039	<0.001
ITA	46.851	<0.001	115.366	<0.001
L*	42.368	<0.001	51.813	<0.001
A*	6.196	<0.001	3.707	0.012
B*	13.083	<0.001	14.646	<0.001

Paired comparisons of rash and bicep skin characteristics

Significant differences were seen in paired samples analysis in EI, melanin, ITA, L*, and A* values (all p<0.001), but not B* (yellow-blue axis) value (p = 0.546). Quantitative differences are shown in Table 4. Significant differences in Melanin, ITA, and L* values suggest that darker pigmentation was higher in rash areas, potentially due to post-inflammatory pigmentation changes associated with the rash.

**Table 4:** Paired comparisons of rash and bicep skin characteristics Paired samples t-test was used to compare baseline (bicep) and rash measurements. Mean Difference (Bicep - Rash); SD = Standard Deviation; EI = Erythema Index; MI = Melanin Index; ITA = Individual Typology Angle; L*a*b* = CIELAB color dimensions (L* = lightness, A* = red-green axis, B* = yellow-blue axis). Cohen’s d values represent paired effect sizes (dz), calculated as the mean difference divided by the standard deviation of the paired differences. Effect size interpretation: small ~ 0.2, moderate ~ 0.5, large ≥ 0.8.

Parameter	Mean difference ± SD	t (df=43)	p-value	Cohen’s d
EI	-29.14 ± 50.3	-3.84	<0.001	0.58
MI	-79.39 ± 78.5	-6.71	<0.001	1.01
ITA	28.77 ± 21.8	8.76	<0.001	1.32
L*	8.48 ± 7.84	7.17	<0.001	1.08
A*	-5.58 ± 6.52	-5.69	<0.001	0.86
B*	0.46 ± 5.0	0.61	0.546	0.09

Correlation analysis

Baseline correlations demonstrated significant relationships among various skin characteristics. A moderate positive correlation was observed between bicep EI and melanin (r = 0.521, p < 0.001), indicating that EI levels tend to increase with melanin content in baseline skin areas. Strong negative correlations were identified between melanin and ITA (r = -0.941, p < 0.001) as well as between melanin and lightness (r = -0.973, p < 0.001), suggesting that increased melanin is associated with lower ITA values and decreased lightness.

**Figure 2:**
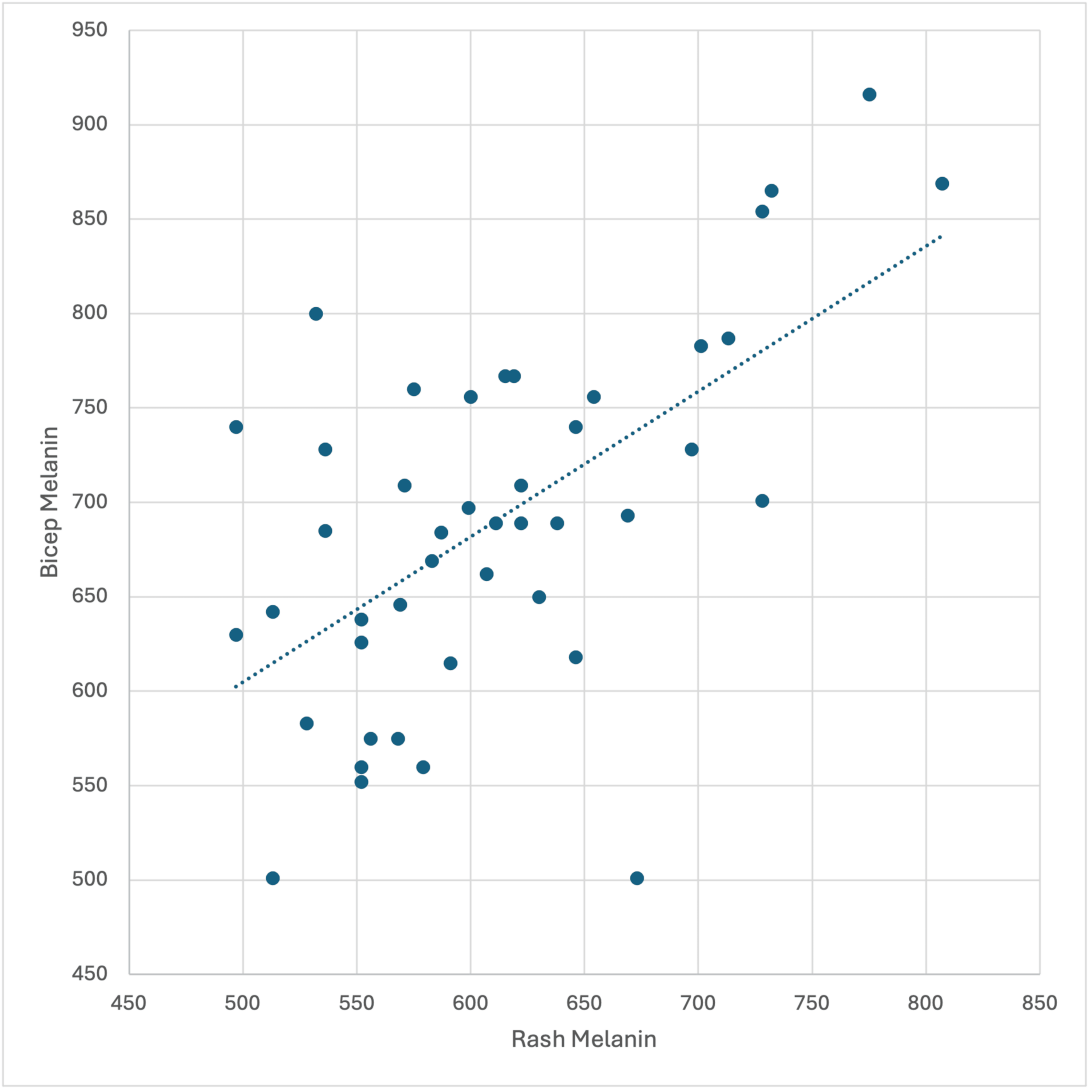
Correlation between rash and bicep melanin Scatter plot showing a moderate positive correlation between rash melanin and bicep melanin (r = 0.602, p < 0.001).

Consistent patterns between baseline and rash areas were also evident. As seen in Figure 2, rash melanin was significantly correlated with bicep melanin (r = 0.602, p < 0.001). In contrast, positive correlations between rash and bicep ITA and between rash and bicep lightness indicated that skin tone and lightness remained relatively stable in both unaffected and affected areas.

**Figure 3:**
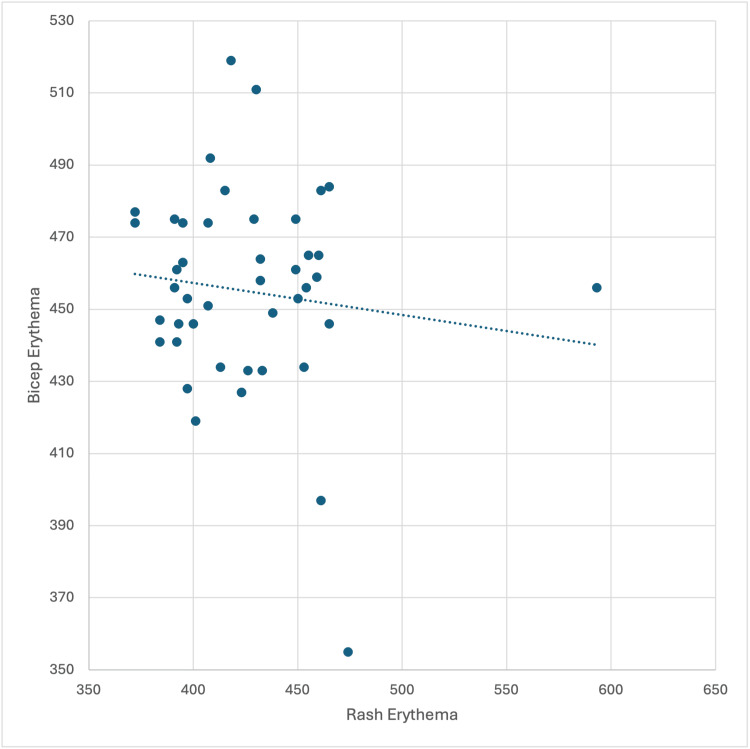
Correlation between rash and bicep erythema Scatter plot showing no significant correlation between rash erythema and bicep erythema (r = –0.156, p = 0.206).

Unexpectedly, there was no significant correlation between bicep and rash EI (r = -0.156; p=0.206), indicating that erythema levels in baseline areas did not predict erythema levels in affected (rash) areas (Figure 3). This lack of association contrasts with the consistent relationships observed for other skin characteristics and highlights a distinct variability in erythema between unaffected and affected regions.

## Discussion

This study examined the correlation between erythema and skin tone in papulosquamous and eczematous conditions using objective colorimetric tools. Results largely reflected expected dermatologic patterns: higher phototypes demonstrated increased melanin with lower ITA and lightness values, and erythema was significantly elevated in rash sites, confirming its association with inflammation. Rash areas also showed increased melanin compared to baseline. This pattern is consistent with prior models in which inflammatory insults are followed by erythema and subsequent pigmentary changes, with post-inflammatory hyperpigmentation more prominent in individuals with higher baseline melanin [12]. Erythema in inflammatory skin conditions is driven by dermal vasodilation and increased blood flow, mediated by inflammatory cytokines, histamine, prostaglandins, and bradykinins [13]. Vascular reactivity and immune activation shape its clinical presentation, and emerging evidence suggests that individuals with darker phototypes may exhibit distinct microvascular responses [13].

Clinically, erythema remains a fundamental component of disease assessment in dermatology, including for psoriasis (PASI), atopic dermatitis (EASI), and rosacea severity scoring. However, multiple studies have shown that visual assessment of erythema is inherently unreliable, especially in patients with darker skin tones. Visual PASI erythema scoring is subjective and prone to inter- and intra-observer variation, even among experienced dermatologists [14]. In addition, the erythema Q-score demonstrated improved reliability compared with clinician assessment, with an intra-class correlation coefficient of 0.84 for the algorithm versus 0.67 for dermatologists and a correlation coefficient of 0.85 between groups [15].

In patients with skin of color, erythema may appear violaceous, gray, or brown rather than classic redness, contributing to underrecognition, misclassification, delayed diagnosis, and underestimation of disease severity across inflammatory dermatoses [16,17]. These challenges have been described across multiple dermatologic conditions, including atopic dermatitis and psoriasis, where erythema-based scoring systems may underestimate disease burden in patients with darker skin tones [18].

In the present study, darker phototypes unexpectedly exhibited higher erythema levels, contrary to longstanding assumptions that erythema is less visible in these populations [19]. This finding highlights a distinction between visual perception and objective quantification, as colorimetric tools can quantify erythema across a range of skin tones within this study population while reducing reliance on subjective visual assessment, although melanin remains an important optical factor influencing measurement [2]. These findings contrast with prior literature suggesting reduced erythema visibility in darker skin tones, in which erythema is often clinically underestimated rather than overestimated [16,17]. While previous studies emphasize diagnostic masking due to melanin, the present results suggest that objective colorimetric measurements may reveal underlying inflammatory changes that are not visually apparent. This distinction highlights the difference between perceptual limitations and measurable physiologic signals, underscoring the importance of incorporating objective tools alongside traditional clinical assessment.

From a mechanistic perspective, this observation is supported by the optical properties of skin. Skin color is determined by the combined contributions of melanin and hemoglobin, whose absorption spectra overlap across the visible light range. Melanin has broad absorption across the visible spectrum and can attenuate hemoglobin-related signals, thereby reducing the visibility of erythema while still permitting detection through reflectance-based measurement systems [20-22]. This phenomenon may help explain how erythema can be objectively elevated despite reduced clinical visibility in darker skin tones.

Instrumental techniques such as colorimetry and erythema-directed digital imaging have been shown to correlate significantly with clinical erythema severity and, in some cases, detect inflammatory changes earlier than visual examination. In rosacea, colorimeter and erythema-directed digital photography demonstrated significant concordance and correlation with clinical grading, including κ = 0.578 and r = 0.637 at baseline, increasing to κ = 0.714 and r = 0.791 at week 2 [23]. Similarly, image-based assessment using CIELAB parameters showed significant reductions in lesional a* and Δa* over time (P = .005 to < .001), with weak but significant correlations with clinical scores (Rs = 0.37 and 0.30) and strong interobserver agreement (R² = 0.82), supporting reproducible objective monitoring despite only modest concordance with clinician grading [24].

These findings collectively suggest that higher erythema measurements observed in darker phototypes in this study may reflect improved instrumental detection of underlying inflammation that is visually underappreciated, rather than true increases in vascular response alone. However, physiologic variation cannot be excluded, as prior work has demonstrated differences in endothelial function and cutaneous blood flow responses across populations [25].

Paired within-subject analyses showed significant increases in erythema at rash sites from baseline, indicating consistent directional changes within individuals. In contrast, correlation analysis revealed no significant association between baseline erythema and rash erythema, indicating that baseline erythema did not predict erythema severity in affected skin across participants. This difference suggests that the statistical significance reflects intra-individual change rather than population. Although baseline and rash measurements had overlapping ranges, such overlap is expected in paired physiological data and reflects differences between individuals rather than a lack of meaningful variation, especially given the moderate-to-large effect sizes observed. Some variables were not strictly normally distributed; however, parametric tests were still used because the sample size exceeded 30 and the primary analyses relied on paired within-subject comparisons, which are considered robust.

In contrast, baseline and rash melanin measurements demonstrated a moderate positive correlation (r = 0.602), indicating greater stability of pigmentation characteristics between unaffected and affected skin. Together, these findings highlight a fundamental difference in measurement behavior: melanin reflects relatively stable, individual-specific traits, whereas erythema reflects localized inflammatory activity that is not predictable from baseline measures. This distinction is supported by prior studies demonstrating that erythema represents a dynamic vascular process influenced by acute inflammatory signaling, whereas pigmentation reflects more stable structural and melanocytic characteristics of the skin [12]. These differences accentuate the importance of interpreting erythema and melanin measurements as distinct but complementary parameters in dermatologic assessment. These patterns do not indicate methodological limitations but emphasize the importance of distinguishing between-subject differences and within-subject changes when interpreting erythema measurements.

These findings support a growing body of literature advocating for objective, pigment-independent assessment tools to have the potential to improve diagnostic accuracy and support more equitable dermatologic care. Current clinical scoring systems remain heavily dependent on erythema as a visual endpoint, despite well-documented limitations in patients with darker skin tones. Incorporation of quantitative measurement techniques may improve disease monitoring across a wide range of dermatologic conditions, including psoriasis, atopic dermatitis, and inflammatory acne, where accurate assessment of erythema is critical for staging disease severity and guiding treatment decisions.

Future research should focus on larger, more representative cohorts and incorporate advanced imaging techniques to determine whether observed differences are biological or methodological. Addressing these issues will be essential for improving erythema detection and ensuring equity in dermatologic care. Given the exploratory, cross-sectional design and limited subgroup representation, these findings should be interpreted as associative and hypothesis-generating.

Study limitations

The study’s small sample size limits its generalizability, especially for individuals with darker skin tones. Additionally, the sample size limits statistical power for subgroup analyses and higher-order interaction modeling, increasing the risk of Type II error for certain comparisons. Rash chronicity and severity were not assessed, which may have affected the results. The distribution of skin phototypes was uneven, with underrepresentation of darker phototypes, which may limit subgroup interpretation. In addition, rash site selection based on the most clinically erythematous region may introduce measurement bias.

Because this study was exploratory and observational in design, a priori power calculations were not performed. While paired analyses reduce inter-individual variability and increase sensitivity to within-subject change, effect size estimates derived here should be interpreted as preliminary. The inclusion of multiple inflammatory dermatoses may introduce spectrum bias, as different conditions exhibit distinct vascular and pigmentary characteristics. Larger, more diverse, and longitudinal studies are needed to confirm whether findings reflect biological variation or device limitations.

## Conclusions

This study provides quantitative evidence on erythema and pigmentation across diverse skin tones using objective colorimetric tools. Confirmed associations among melanin, ITA, and lightness support the reliability of these measures. Notably, erythema in rash areas did not correlate with baseline erythema, and higher erythema values were observed in darker ITA categories, suggesting that localized inflammation may surpass baseline skin tone effects and that traditional methods may underestimate erythema variation.

Together, these results underscore the need for improved erythema detection methods that accommodate all skin tones. Future research should use larger, more diverse cohorts and longitudinal designs to better characterize erythema and pigmentation changes during treatment, distinguishing physiological differences from measurement limitations. Enhanced assessment will improve diagnostic accuracy and promote equitable dermatologic care by shifting away from solely visually dependent frameworks toward standardized, quantitative approaches that account for variability in skin pigmentation, with direct implications for more accurate disease severity assessment, treatment evaluation, and monitoring across inflammatory dermatoses.
